# Spheno-occipital synchondrosis as a reliable indicator of skeletal maturity: A systematic review and meta-analysis

**DOI:** 10.34172/joddd.025.41168

**Published:** 2025-03-31

**Authors:** Swati Singh, Ravindra Kumar Jain, Niha Naveed, Arthi Balasubramaniam

**Affiliations:** ^1^Department of Orthodontics, Saveetha Dental College and Hospitals, Saveetha Institute of Medical and Technical Sciences, Chennai, Tamil Nadu, India; ^2^Department of Public Health Dentistry, Saveetha Dental College and Hospitals, Saveetha Institute of Medical and Technical Sciences, Chennai, Tamil India

**Keywords:** Cervical vertebral maturation, Growth, Hand-wrist radiographs, Maturation, Spheno-occipital synchondrosis

## Abstract

The present review systematically analyzed the available literature on the application of spheno-occipital synchondrosis (SOS) as a skeletal maturity indicator in growing subjects reporting for orthodontic treatment. A systematic search of the available literature on SOS for assessment of skeletal maturity was performed in various databases like Google Scholar, PubMed, and Scopus until December 2023. The risk of bias assessment was performed using the STARD (Standards for the Reporting of Diagnostic Accuracy Studies) statement. A narrative summary on the correlation of SOS with cervical vertebral maturation index (CVMI), hand-wrist maturation (HWM), and chronologic age was done, followed by a meta-analysis with a fixed effects model to quantitatively assess the correlation of SOS with CVMI. A total of eight studies were included in the review, and seven of them reported a significant, strong positive correlation between SOS and CVMI, two studies reported a moderate correlation of SOS with HWM stages, and seven studies reported a good to moderate correlation between SOS and chronologic age. Most included studies had a low risk of bias and were considered good-quality studies. On performing the meta-analysis of both the 4- and 5-stage SOS, a significant positive correlation of SOS with CVMI was noted (*P*<0.05). The present high-quality evidence suggests a strong positive correlation between SOS and CVMI, a moderate correlation between SOS and HMW, and a moderate correlation between SOS and chronologic age. Hence, SOS can reliably be used as a skeletal maturity indicator.

## Introduction

 Timing for treatment intervention in orthodontics is very important, especially for growth modulation with functional and orthopedic appliances, as it significantly affects the outcome.^1,2^ There are various ways to determine the optimal time and assess the patient’s potential for growth. The most conventional and predictable method is hand-wrist maturation (HWM), which entails irradiation exposure. The other widely used skeletal maturity indicator is cervical vertebral maturation (CVM), which is strongly associated with the hand-wrist approach and is regarded as a very reliable technique for evaluating the skeletal maturation of the mandible.^3-5^

 The three synchondroses in the cranial base are significant growth centers, including spheno-ethmiodal synchondrosis, inter-sphenoid synchondrosis, and spheno-occipital synchondrosis (SOS). A cartilaginous connection between the sphenoid body and the basilar portion of the occipital bone is known as SOS. The maxilla is carried upward and forward about the mandible by growth at the SOS, increasing the depth and height of the face. Studies have demonstrated that a protracted orthopedic force on the maxilla induces chondrogenic processes in SOS, and the effectiveness of these appliances is influenced by the fusion stages of SOS. One study concluded that the SOS maturation phases were reliable and accurate markers of skeletal maturity for maxillary growth. Hence, the assessment of SOS staging is important in the planning and execution of orthopedic treatment targeting the maxilla.^6-8^

 Of late, 3D imaging has become widely used in dentistry and orthodontics to diagnose, plan, and assess different treatment modalities and their outcomes. SOS evaluation can only be carried out using CBCTs as they are not easily seen on 2D radiographs.^9,10^ Studies on the correlation of SOS staging with HWM, CVM, and chronological age have been reported, but no systematic reviews are available. This review was performed to address the question of whether SOS staging is as effective as the other skeletal maturity indicators for determining the pubertal growth spurt.

## Methods

###  Protocol and registration 

 The PRISMA 2020 statement reporting standards for systematic reviews and meta-analyses were followed in the reporting in this review. This systematic review was registered under the CRD42023448412 registration number and submitted to the PROSPERO database of the International Prospective Register of Systematic Reviews.

###  Search strategy 

 Without applying any limitations on publication date or language until December 2023, an electronic search of the literature in the databases listed below—PubMed, Scopus, and Google Scholar—was conducted to find all the articles on the study topic. Table 1 includes the number of search papers in each database and the keywords used. Grey literature was searched using Open Grey and GreyNet International. Rayyan.ai’s duplicate removal tool was used to eliminate duplicates.^11^

**Table 1:** Keywords and databases searched

**Database **	**Keywords**	**No.**
PubMed	(((((((((((((((spheno occipital synchondrosis maturation) OR (spheno occipital synchondrosis stages)) OR (basi occiput)) OR (basicranial))) AND (growth assessment)) OR (growth prediction)) OR (puberty)) OR (pubertal growth spurt assessment)) AND (correlation)) OR (reliability)) AND (cervical vertebrae maturation)) OR (cervical vertebrae)) OR (cervical maturation assessment)) AND (hand wrist maturation index)) OR (hand wrist radiograph skeletal maturity indicator)	139
Scopus	spheno AND occipital AND synchondrosis AND maturation AND growth AND assessment OR growth AND prediction	64
Google Scholar	sphenooccipital synchondrosis maturation AND growth assessment AND correlation AND cervical vertebrae maturation stages AND hand wrist radiographs	211

###  Eligibility criteria

 The papers to be included in the review were selected based on the eligibility criteria in PICO format. Studies on human subjects were included in which growth status or pubertal growth spurt of both males and females was assessed using SOS maturation stages or SOS maturation index (3-, 4-, 5-, or 6-stage SOS evaluation), and control comparison was made with other skeletal maturity indicators like CVM stages or HWM stages or dental age assessment and outcomes assessed were correlation or reliability of SOS maturation with other skeletal maturity indicators. Both prospective and retrospective studies were included. Studies on the correlation of SOS maturation only with chronologic age and maxillary and mandibular growth were excluded.

###  Screening and selection of studies

**Figure 1:**
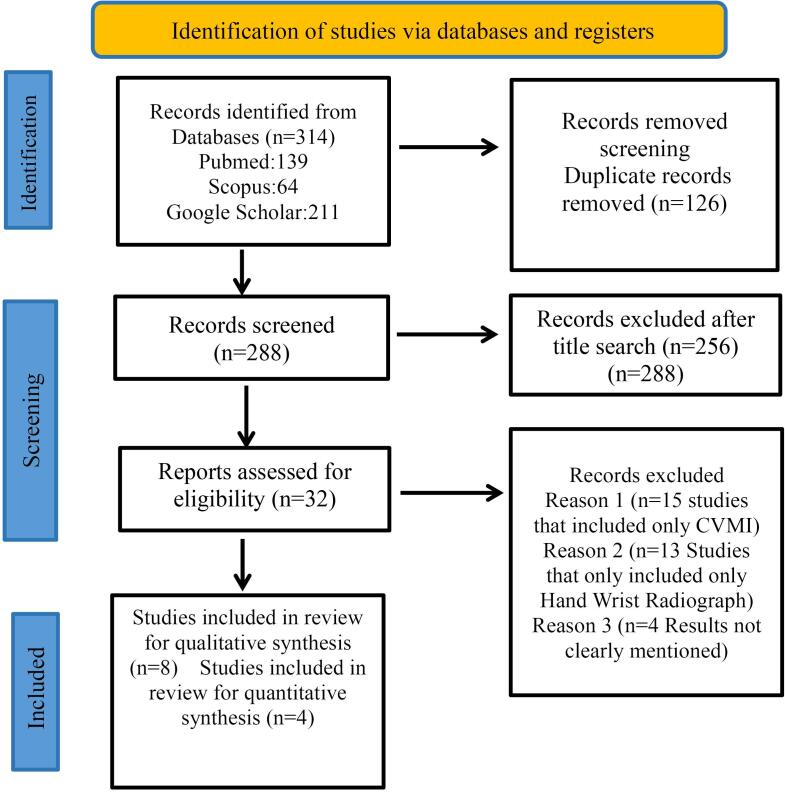
Prisma flow chart illustrating the method used to find included studies

 Authors SS and RKJ screened the titles and abstracts individually, and after mutual consensus, all studies meeting the selection criteria were included in the review. The selection process of included studies is depicted in the PRISMA flow chart (Figure 1). Data required for analysis were extracted independently by both reviewers (SS and RKJ). Study characteristics from the included articles were tabulated and included information about the first author and year of publication, study design, sample size, intervention, control groups, statistics used, outcomes measured, and the individual study conclusions. Any disagreements between the authors (SS and RKJ) regarding data collection were handled by mutual discussion and resolved by consulting a third author (AB).

###  Risk of bias assessment

 Using the tools “Strengthening the Reporting of Observational Studies in Epidemiology” (STROBE) and Standards for the Reporting of Diagnostic Accuracy Studies (STARD), the risk of bias in each study was assessed.^12,13^ The authors (SS and RKJ) completed the ROB assessment. When the two reviewers could not agree, a third investigator (AB) was contacted in an attempt to achieve a consensus. Each criterion was assigned a point if satisfied and no points if not satisfied. Quality assessment scores ranged from 0 to 12. Articles were classified as “low quality” (score from 0 to 6), “moderate quality” (score from 7 to 10), or “high quality” (score from 11 to 12) (Table 2).

**Table 2:** STROBE guidelines for ROB assessment

		**Yes**	**No**
1	Is the objective clearly formulated?	1	0
2	Are there key elements of study design early in the paper?	1	0
3	Was the sample size calculated?	1	0
4	Does the study report the demographic characteristics of the study population?	1	0
5	Were the sample selection criteria clearly described?	1	0
6	Does the study describe the specifications of the material and methods involved including how and when measurements were taken?	1	0
7	Was there a reliability assessment, with an adequate level of agreement intraexaminer or/and interexaminer?	1	0
8	Were measurements undertaken blindly?	1	0
9	Does the study give details of methods of assessment (measurements) for each variable of interest?	1	0
10	Was there a complete and adequate reporting of results, with self-explanatory tables and figures?	1	0
11	Was there a statistical analysis appropriate for data?	1	0
12	Was the *P* value stated or confidence intervals provided?	1	0

###  Synthesis measures

 The studies included in this review employed different methods of SOS staging in different populations. A fixed-effects meta-analysis was performed to assess the correlation of 4-stage SOS and 5-stage SOS with cervical vertebral maturation index (CVMI). A qualitative summary of the correlation of SOS with CVMI, HWM, and chronologic age was performed, illustrating the results of individual studies based on the groups evaluated.

###  Outcome measures

 The outcome measures of this review included the correlation between SOS maturation stages and other skeletal maturity indicators like CVMI and HWR, as well as the intra- and inter-rater correlation for SOS staging.

## Results

###  Study selection based on PRISMA flowchart

 The initial search of all databases yielded 414 studies, and after removing duplicates manually, the remaining 288 studies were subjected to title screening. A total of 256 papers were excluded, and 32 studies were subjected to abstract screening for the eligibility criteria. Finally, eight studies^14,15,16-21^ were included for qualitative analysis and four for quantitative synthesis.^15,18,19,21^

###  ROB assessment

 Except for the study by Alhazmi et al,^17^ which was of moderate quality, all the included studies were of high quality. Sample size calculation was not reported in 6 studies.^14-18^ Outcome measurement blinding was not reported by Alhazmi et al (Table 3).

**Table 3:** ROB assessment of the included studies based on STROBE and STARD guidelines

**ROB ** **criteria**	**DemirturkKocasarac et al**^15^	**Halpern**^14^	**Fernández-Pérez et al**^21^	**Fayad et al**^19^	**Dillon**^16^	**Alhazmi**^17^	**Fu**^20^	**Kim et al**^18^
1	1	1	1	1	1	1	1	1
2	1	1	1	1	1	1	1	1
3	0	0	1	0	0	0	1	0
4	1	1	1	1	1	1	1	1
5	1	1	1	1	1	1	1	1
6	1	1	1	1	1	1	1	1
7	1	1	1	1	1	1	1	1
8	1	1	1	1	1	0	1	1
9	1	1	1	1	1	1	1	1
10	1	1	1	1	1	1	1	1
11	1	1	1	1	1	1	1	1
12	1	1	1	1	1	1	1	1

###  Qualitative assessment of the included studies 

 Out of the eight studies included in the review, seven were high-quality studies^14,15,16,18-21^ and reported on the correlation between SOS and CVMI, with two studies reporting on the correlation between SOS and hand-wrist assessment.^16,19^ The correlation between dental age and SOS was reported in two studies.^14,15^ All the studies reported using correlation tests and the intra- and inter-rater reliability. The age range of the included subjects was 6‒30 years. Different methods of SOS staging were used in the studies included, and all the included studies employed CBCT for imaging the SOS. The five-stage SOS scoring method by Bassed et al^22^ was used in three studies.^20,21^ The four-stage SOS method by Franklin and Flavel^23^ was employed in two studies.^15,19^ The three-stage SOS scoring method was used in two studies.^14,17^ (Tables 4 and 5)

**Table 4:** Study characteristics

**Author, Year**	**Study sample**	**Intervention**	**Comparator**	**SOS reliability**	**SOS correlation stats**
Halpern, 2014, retrospective study^14^	CBCTs of 77 subjects (42 F & 35 M, age 9-21 years) divided into 6 groups	SOS maturation three-stage index by Melsen (1972)	1. CVM index by Baccetti 2. DI 3. Chronologic age	Cohen kappa	Spearman correlation
Demirturk Kocasarac et al, 2016, retrospective study^15^	Panoramic radiographs, lateral cephalometric radiographs and CBCT scan 116 subjects (43 M and 73 F, age 8-18 years)	Assessment of SOS maturation using the four-stage system by Franklin and Flavel^24^	1. CVM assessment by Hassel and Farman 2. Dental age estimation with third molar maturation (TMM) 3. Chronologic age	Kappa stats for intraobserver	Spearman correlation
Fernández-Pérez et al, 2016, retrospective study^21^	CBCTs of a 315 participants (167 M and 148 F, age 6-23 years)	Assessment of SOS maturation using the 5 stage system by Bassed et al^23^	CVM index by Baccetti	Cohen kappa stats for intra and inter-rater agreement	Spearman non-parametric tests
Dillon, 2018, retrospective study^16^	CBCT and HW radiograph taken within three months of each other. 61 patients, 33 F and 28 M, Age 9.5-17.5 years	Analysis of SOS maturation by Kinder's five-stage method (2009)	1. CVM index by Baccetti. 2. HWM by Hagg and Taranger	kappa statistics and Kendall’s coefficient of concordance	Spearman correlation
Fu, 2018, retrospective study^20^	CBCT scans of 275 patients aged 6 to 30 years	Assessment of SOS maturation using a 5 stage system by Bassed et al^23^	1. CVM index by Baccetti.	Cohen kappa	Spearman correlation *P* value < 0.05
Fayad et al, 2020, retrospective study^19^	CBCT scans and lateral cephalograms of 117 patients (55 M and 62 F, age 8-18 years)	Assessment of SOS maturation by the 4 stage method of Franklin and Flavel^24^	1. CVM index by Baccetti 2. Measurements of ANB and GoGn-SN angles	Cohen’s Kappa observer reliability	Spearman correlation Fisher exact test
Alhazmi et al, 2021, retrospective study^17^	CBCT scan and HW radiographs of 164 subjects (77 M and 87 F, age 10-18 years)	Assessment of SOS maturation using a modified three-stage system	HWM index by Fishman	Cohen’s kappa observer reliability tests	Kendall rank correlation fisher exact tests for association of HWM and SOS stages
Kim et al, 2023^18^	CT images of 630 Korean subjects in the age range of 6-18 years	5 stage SOS scoring system	1. CVM index by Baccetti 2. Chronologic age	Kappa coefficient	Spearman’s correlation analysis

DI: development of the mandibular canine; CBCT: cone-beam computed tomography; CVM: cervical vertebrae maturation; SOS: spheno-occipital synchondrosis; CT: computed tomography; HWM: hand-wrist maturation: M: males; F: females

**Table 5:** Results of the included studies

**Author, Year**	**Results **	**Inference**
Halpern, 2014, retrospective study ^14^	SOS maturation showed a significant correlation with both chronologic age (0.830 F and 0.849 M and CVM (0.831 F and 0.870 M (*P* ≤ 0.001). A weak correlation (*P* ≤ 0.001) was discovered between SOS maturation and DI (0.734 F and 0.638 M).	CVM corresponds with SOS stages.
Demirturk Kocasarac et al, 2016, retrospective study^15^	For both genders, there is a strong correlation (r = 0.810) between age and SOS fusion (r = 0.643). TMM and SOS were shown to have strong (r = 0.759) and moderate (r = 0.534) correlations for males, respectively. CVM and SOS fusion showed positive and good (r = .851) and strong (r = 0.618) correlations for females.	In the young Turkish population, a good correlation between the degrees of TMM, fusion of SOS, and CVM.
Fernández-Pérez et al, 2016 retrospective study^21^	Age and SOS had a strong correlation (r = 0.66). Additionally, sex and SOS had a weak but significant correlation (r = 0.15). Strong and substantial correlations were found between vertebrae and SOS (r = 0.89), age and synchondrosis (r = 0.73, and r = 0.65, for females and males respectively, *P* < 0.001), and both for females and males.	From the SOS stage, the CVM stage may be accurately predicted. The SOS stages are a reliable indicator of maturation of growth.
Dillon, 2018, retrospective study^16^	Average intra and inter-rater reliability was noted for SOS (intra-rater ĸ = 0.642; inter-rater ĸ = 0.886). A moderate correlation between CVMI and SOS (r = 0.68, spearman correlation), and the kappa coefficient - 0.25, which indicates fair agreement. HWM vs SOS correlation was moderate (r = 0.73) The SMIs that agreed most closely were the HWM and SOS (ĸ = 0.5079). Chronologic age was not well correlated with SOS.	SOS stages had least intra and inter-rater agreement. SOS closure was not well correlated with age and had the highest agreement with HWM.
Fu, 2018, retrospective study^20^	A strong positive correlation between the CVM and SOS closure (*rs*= 0.908) and between SOS stages and age was noted (*rs*- 0.857)	The maturation stage of SOS is a potential indicator for skeletal maturity assessment
Fayad et al, 2020, retrospective study ^19^	SOS maturation and CVM were shown to be strongly correlated (r = 0.852, *P* < 0.001 spearman correlation, females - 0.86, males - 0.83). Only in the CVM stage 2 group and in the CVM stage 4 group did gender and SOS significantly correlate, according to Fisher's exact test. For the SOS index, Cohen's kappa analysis revealed nearly perfect intra-rater reliability (k = 0.85, *P* < 0.001) and strong inter-rater reliability (k = 0.80, *P*< 0.001). Strong correlations were seen between chronological age and SOS fusion (r = 0.705, *P* < .001), with males showing higher correlations (r = 0.819, *P*< 0.001) than females (r = 0.794, *P* < 0.001).	CVM and SOS fusion have good correlation. Compared to males, girls have an earlier fusion of SOS to their CVM, and the vertical growth pattern may have an impact on the association between SOS and CVM stages.
Alhazmi et al, 2021, retrospective study ^17^	For both genders, a strong positive correlation between HWM maturity and SOS fusion stages was found. In both males and females, Kendall's rank correlation between HWM and SOS was high and positive (r = 0.74 and r = 0.71, respectively).	A biological marker for mandibular and craniofacial growth spurt prediction is SOS fusion.
Kim et al, 2023 ^18^	In both males and females, there was a significant correlation (*P* < 0.001) between age and the SOS fusion stage (rs = 0.887, rs = 0.885) and between the CVMI stage and the SOS fusion stage (rs = 0.955, rs = 0.964).	Basic references for the CVMI stage and the SOS fusion stage from preadolescents to young adults in the Korean population has been provided.

DI: development of the mandibular canine; CBCT: cone-beam computed tomography; CVM: cervical vertebrae maturation; SOS: spheno-occipital synchondrosis; CT: computed tomography; HWM: hand-wrist maturation; M: males; F: females

###  Association of SOS with CVMI

 Seven studies evaluating the correlation between SOS and CVMI reported a significant positive correlation (*P* < 0.001) in both genders and used either Spearman’s or Pearson’s correlation. Six out of the seven studies reported a strong correlation,^14,15,18-21^ whereas one study^16^ reported a moderate correlation in both males and females. All the studies found that the beginning of SOS closure coincided with the onset of PHV, and a complete closure of SOS indicated the completion of the pubertal growth spurt.

###  Quantitative assessment of 4-stage SOS with CVM

**Figure 2:**
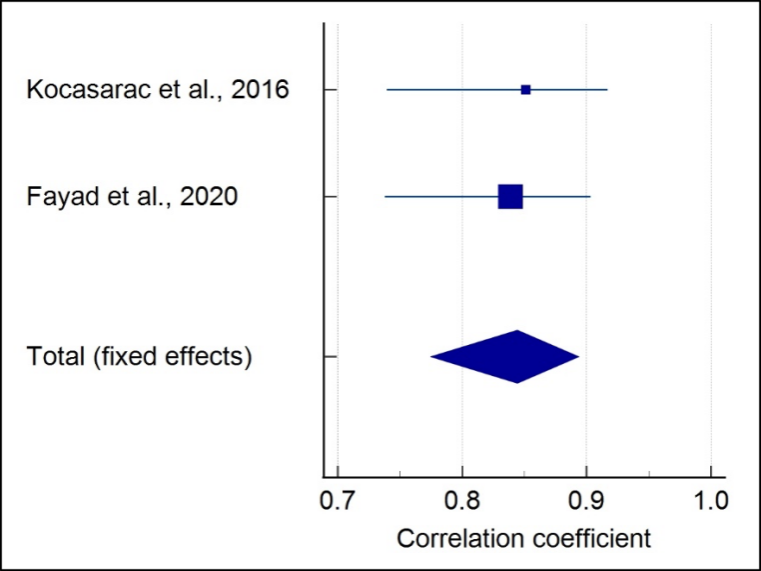
Forest plot of correlation between 4 stage SOS and CVMI in males

**Figure 3:**
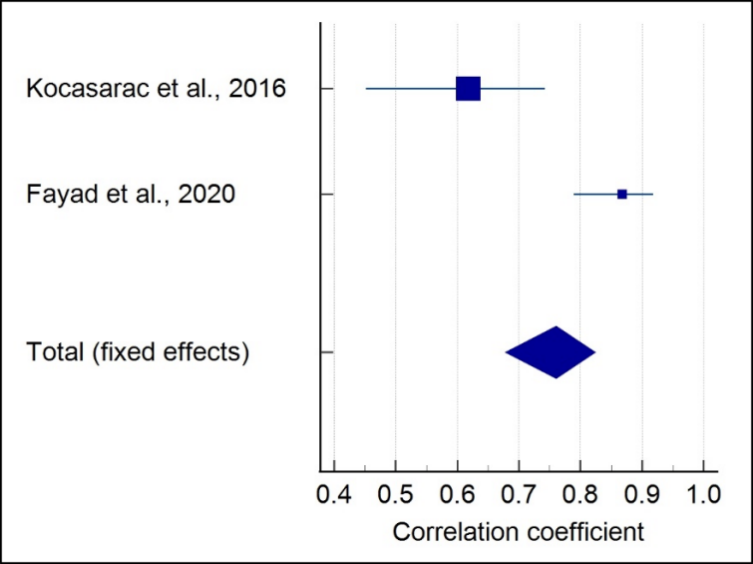
Forest plot of correlation between 4-stage SOS and CVMI in females

**Figure 4:**
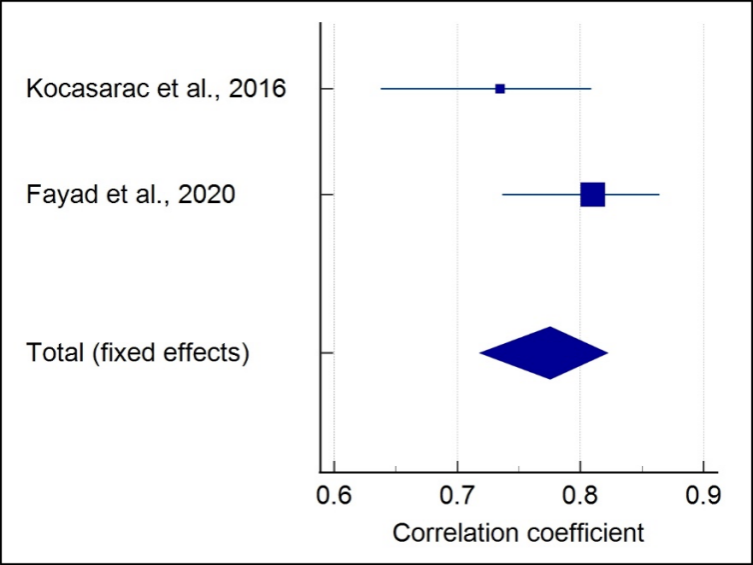
Forest plot of correlation between 4 stages SOS and CVMI for no gender discrimination

 Meta-analysis of the correlation between the 4-stage SOS and CVM showed that each included study showed a significant strong positive correlation in males (r = 0.844) (*P* < 0.001) (Figure 2, Table S1), and no heterogeneity was reported (I^2^ = 0%). A moderate positive correlation (r = 0.761) (*P* < 0.001) with high heterogeneity (I^2^ = 91.41%) was noted in females (Figure 3, Table S2). A moderate positive correlation (r = 0.775) (*P* < 0.001), with a moderate heterogeneity (I^2^ = 49.9%) (Figure 4, Table S3), was noted when gender was not considered.

###  Quantitative assessment of 5-stage SOS with CVM

**Figure 5:**
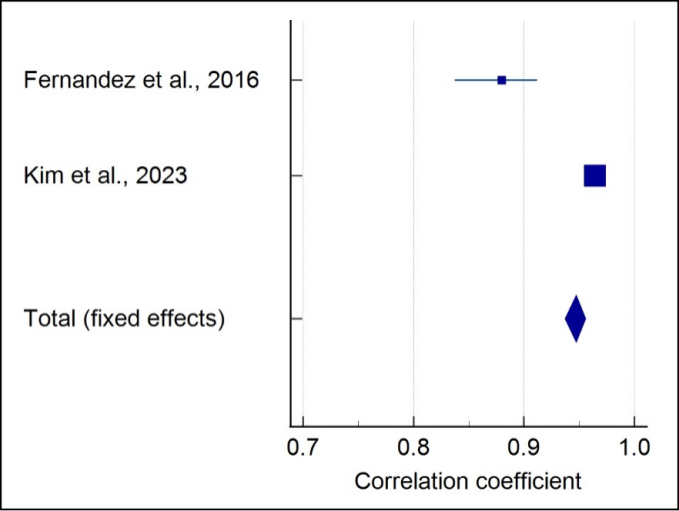
Forest plot showing the meta-analysis correlation coefficient results for CVM and SOS in female gender (5 stage)

**Figure 6:**
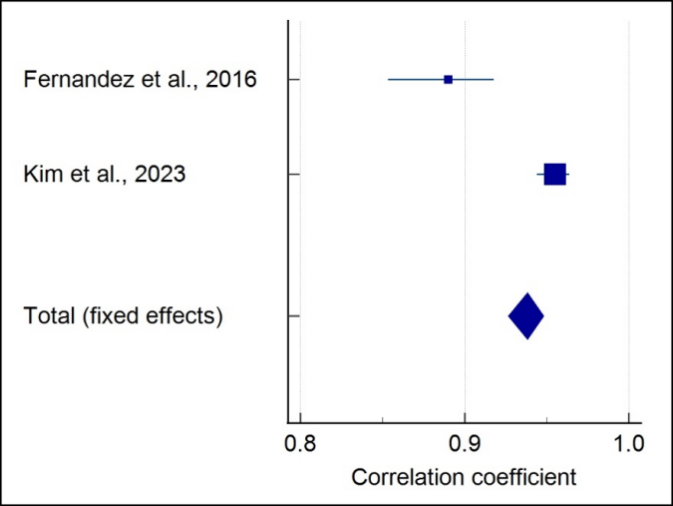
Forest plot showing the meta-analysis correlation coefficient results for CVM and SOS in male gender (5 stage)

**Figure 7:**
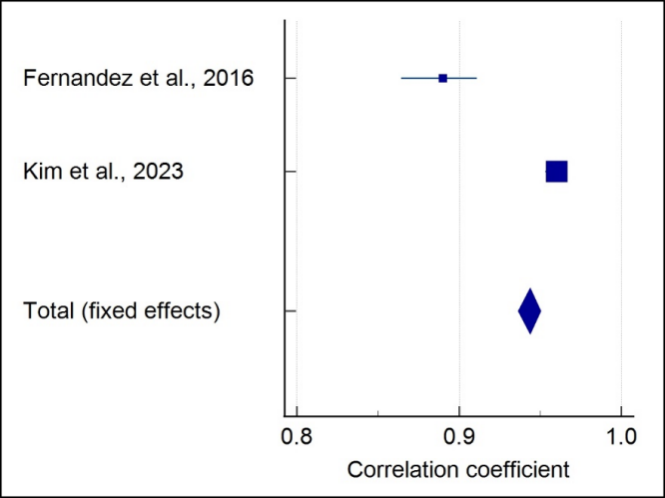
Forest plot showing the meta-analysis correlation coefficient results for CVM and SOS with no gender discrimination (5 stage)

 Meta-analysis of the correlation between the 5-stage SOS and CVM stages showed that each included study showed a significantly strong positive correlation in males (r = 0.938) (*P* < 0.001) (Figure 5, Table S4), and a high heterogeneity was reported (I^2^ = 95.64%). A strong positive correlation in females (r = 0.947) (*P* < 0.001) with high heterogeneity (I^2^ = 97.42%) was noted (Figure 6, Table S5). A strong positive correlation (r = 0.944) (*P* < 0.001), with high heterogeneity (I^2^ = 98.25%) (Figure 7, Table S6) was noted when gender was not considered.

###  SOS association with HWM 

 Correlation between HWM and SOS fusion percentage was significant and moderate to good in both males and females, as noted in both studies.^16,17^ Meta-analysis could not be conducted as both included studies employed different methods for SOS and HWM staging.

###  Association with dental development

 A weak correlation was found between SOS maturation and dental development (DI), as reported by Halpern,^14^ and a moderate association in females and a strong correlation in males was noted by Demirturk Kocasarac et al.^15^

###  Correlation with chronologic age

 Except for the study by Alhazmi et al,^17^ all the studies reported the correlation of chronologic age with SOS maturation. One study concluded that SOS closure was not well correlated with age. The other six studies concluded that chronologic age was significantly correlated with SOS.^14,15,18-21^ In general, males had higher correlation values than females.

###  Reliability

 Excellent reliability of the SOS index for both inter- and intra-rater was noted in seven studies^14,15,17-21^ and moderate in one study.^16^

## Discussion

###  Description of the included studies

 The eight included studies were retrospectively performed. They evaluated the potential of SOS as a skeletal maturity indicator. The synthesis of the findings from the included studies provides a fair understanding of the potential use of SOS as a skeletal maturity indicator, its reliability, and the clinical implications of SOS assessment. The studies evaluated different populations and used various methods of SOS assessment. SOS staging correlated well with both CVMI staging and moderately with the HW method of skeletal maturity assessment, irrespective of the method of SOS staging employed, and all the included studies were of high quality with a very minimum risk of bias. Also, a quantitative assessment revealed a significant positive correlation of SOS with CVM staging.

 In the present review, the included studies employed different methods of SOS assessment; hence, not all studies were analyzed quantitatively. Data from two studies of 4-stage SOS assessment^15,19^ and two studies for 5-stage SOS assessment^21^ were pooled separately, and a meta-analysis was performed. Meta-analysis was performed separately for males and females as the correlation values varied. It was observed that for the 4-stage SOS method in males, the highest positive correlation with no heterogeneity was noted compared to a moderate positive correlation with high heterogeneity in females. The meta-analysis showed a high positive correlation with high heterogeneity of the 5-stage SOS with the CVM method in both males and females. Even when gender was not considered, a strong positive correlation with high heterogeneity was observed.

 The SOS has become increasingly significant for age estimation because of its late ossification process, ease of location, and ability to be detected using CBCT imaging to establish its stage of development.^21^ If CBCT is available, an additional HW radiograph is not required to confirm the growth status after a CVM assessment. SOS is essential for the growth of the cranial base and helps define its final shape and how it relates to the upper and lower jaws. Pubertal onset and SOS closure are significantly correlated, and the developing adolescent’s hormonal and systemic changes have an impact on the closure of SOS. The greatest rate of facial growth may coincide with the commencement of SOS closure.^15^ A prior study found that the SOS maturation stages are both a valid and reliable indicator of mandibular growth as measured by three-dimensional cephalometric mandibular measurements,^24^ as well as valid and reliable indicators of maxillary skeletal maturation and three-dimensional maxillary growth in both genders.^8^ It was observed in previous studies that girls tend to have an earlier fusion of the SOS compared to boys in terms of their cervical vertebral stage (CVS), even though this difference was not always significant.^15,19,25-27^ SOS can be considered an alternative to CVMI in subjects with anomalies of the vertebrae and a history of trauma in the cervical region.

 It was noted in the included studies that both males and females have an open SOS at CVS 1. The SOS in girls starts to close at CVS 2, while in boys, the closure would begin at CVS 3. At CVS 4 and 5, the SOS would be closing for both genders and would reach the end of its fusion at CVS 6.^19^ Concerning the frequency between SOS and CVMI, the SOS tends to cluster at later stages, while CVM is spread out but focuses on earlier stages.^17^ Pubertal growth spurt has been reported to coincide with CS4, which corresponds to SOS closure stage 3. Similarly, the beginning of closure of the SOS (Stage 1) coincides with the onset of the pubertal growth spurt.^20,21^ Patients who had a complete union of their SOS were either undergoing or had passed their PHV.^14^

 The studies included in the review were all retrospective, carried out in different populations, and had a very low risk of bias. Convenience sampling was used in most studies, and sample size calculation was performed in only a few of them. All papers included in the review reported a good correlation of SOS with CVM and HWM stages in both genders, but the correlation of SOS with chronologic age varied in both genders. Although recent systematic reviews have shown that the CVM approach can be an effective method for evaluating the growth spurt in growing patients, HW radiography has been considered the gold standard for assessing skeletal maturity.^4,25^ The CVM method’s reproducibility on lateral cephalograms is good, but some studies have found low to moderate reproducibility. However, the CVM method demonstrates satisfactory accuracy and repeatability when precise guidelines are provided along with specific training for visually assessing each stage.^28^ However, when viewed on a CBCT, the current SR’s SOS staging showed good intra- and inter-rater reliability; as a result, SOS can be considered a potential skeletal maturity indicator.

## Limitations

 Some studies had limited sample sizes or age ranges, affecting the generalizability of their findings to broader populations. Differences in methodologies, including SOS assessment systems and imaging modalities, can introduce variability in the results and limit direct comparisons. Very few studies on the correlation of SOS with HWM were available; hence, a quantitative synthesis could not be performed.

## Conclusion

 Considering the limitations of the review, it was observed that high-quality evidence supports the role of SOS as a reliable maturity indicator. SOS stages correlate very well with CVMI and moderately with HWM and hence can be routinely used to assess skeletal maturation.

## Competing Interests

 None declared by the authors.

## Ethical Approval

 SRB/SDC/ORTHO-2103/23/012, Scientific Review Board- Saveetha Dental College.

## Supplementary Files

Supplementary file 1 contains Tables S1-S6.
