# Supplementary material for: Spheno-occipital synchondrosis as a reliable indicator of skeletal maturity: A systematic review and meta-analysis
Source: J Dent Res Dent Clin Dent Prospects. 2025 Mar 31;19(1):1–8. doi: 10.34172/joddd.025.41168 (PMC12128205; doi:10.34172/joddd.025.41168)
Supplement: Supplementary file 1 — contains Tables S1-S6. [file joddd-19-1-s001.pdf]

Singh et al, *J Dent Res Dent Clin Dent Prospects.* 2025;19(1):1-S1
doi: 10.34172/joddd.025.41168

https://joddd.tbzmed.ac.ir

Table S1: Meta-analysis: Correlation between 4-stage SOS with CVMI in males

Study	n	r	Confidence Interval 95%
Kocasarac et al., 2016 [15]	43	0.851	0.740 to 0.917
Fayad et al., 2020 [20]	55	0.839	0.739 to 0.903
Meta-analysis	98	0.844	0.775 to 0.894

Table S2: Meta-analysis: Correlation between 4-stage SOS with CVMI in females

Study	n	r	Confidence Interval 95%
Kocasarac et al., 2016 [15]	73	0.618	0.452 to 0.742
Fayad et al., 2020 [20]	62	0.868	0.789 to 0.919
Meta-analysis	135	0.761	0.678 to 0.824

Table S3: Meta-analysis: Correlation between 4-stage SOS with CVMI in females

Study	n	r	Confidence Interval 95%
Kocasarac et al., 2016 [15]	116	0.735	0.638 to 0.809
Fayad et al., 2020 [20]	117	0.810	0.737 to 0.864
Meta-analysis	233	0.775	0.718 to 0.822

Table S4: Meta-analysis results for correlation between CVM and SOS in females (5-stage)

Study	n	r	Confidence Interval 95%
Fernandez et al., 2016 [22]	148	0.880	0.838 to 0.912
Kim et al., 2023 [19]	322	0.964	0.955 to 0.970
Meta-analysis	470	0.947	0.937 to 0.956

Table S5: Meta-analysis results for correlation between CVM and SOS in females (5-stage)

Study	n	r	Confidence Interval 95%
Fernandez et al., 2016 [22]	167	0.890	0.853 to 0.918
Kim et al., 2023 [19]	308	0.955	0.944 to 0.964
Meta-analysis	475	0.938	0.926 to 0.948

Table S6: Meta-analysis results for correlation between CVM and SOS in females (5 Stage)

Study	n	r	Confidence Interval 95%
Fernandez et al., 2016 [22]	315	0.890	0.865 to 0.911
Kim et al., 2023 [19]	630	0.960	0.953 to 0.966
Meta-analysis	945	0.944	0.936 to 0.950
